# Perfectionism and Anxiety: A Paradox in Intellectual Giftedness?

**DOI:** 10.1371/journal.pone.0041043

**Published:** 2012-07-30

**Authors:** Jacques-Henri Guignard, Anne-Yvonne Jacquet, Todd I. Lubart

**Affiliations:** 1 Centre National d’Aide pour enfant et adolescent à Haut Potentiel, Centre Hospitalier Guillaume Régnier, Rennes, France; 2 Laboratoire Adaptation Travail Individu, Université Paris Descartes, Paris, France; 3 Laboratoire Psychologie de la Perception, Université Paris Descartes, Paris, France; University of Minnesota, United States of America

## Abstract

Numerous authors reported a prevalence of perfectionism in gifted populations. In addition, an unhealthy form of perfectionism that leads to anxiety disorder has been described. Using self-report measures (CAPS and R-CMAS) with 132 children, we hypothesized that intellectually gifted children express a higher level of perfectionism and anxiety. Our results pointed out a paradox: the gifted group obtained a higher self-oriented perfectionism score than the control group in 6th grade, but present the same level of anxiety. In contrast, the gifted group showed the same level of perfectionism than non-gifted 5^th^ graders, but reported a higher anxiety level. Thus, the interplay between perfectionism and anxiety appears to be more complex than a simple linear relationship in giftedness.

## Introduction

Perfectionism is viewed as a specific mode of functioning that corresponds to a tendency to seek to be or to appear perfect. Todorov and Bazinet conceive perfectionism as a personality characteristic [1] and healthy perfectionism has been distinguished from neurotic perfectionism [2]. In the first case, the individual is able to define realistic objectives and gain satisfaction after having reached these objectives. In the second case, the individual fixes excessively high standards of achievement. Because these personal standards are objectively unreachable, they are associated with the uncomfortable feeling that what has been accomplished is incomplete or imperfect. More recently, perfectionism has been viewed as a multidimensional personality trait related to psychological difficulties, distortions of interpersonal relations and an erroneous relationship to success [3]. According to this model, perfectionism is a three dimensional construct including self-oriented, other-oriented and socially-prescribed perfectionism. Self-oriented perfectionism reflects one’s tendency to define high or unreachable personal standards of achievement. It is linked to various traits and disorders including depression, anxiety and hypomania. Other-oriented perfectionism concerns individuals who have high expectations for those in their social environment. Socially-prescribed perfectionism is related to perceived environmental pressures. Socially-prescribed perfectionists perceive pressure from others to hold excessively high standards of achievement. Authors have reported some findings on psychological distress induced by two forms of perfectionism [4]. They collected data from 114 adolescents (10 to 14 years old) using the Children and Adolescent Perfectionism Scale [5], an auto-evaluation scale that measures self-oriented perfectionism and socially-prescribed perfectionism. The results showed correlations between self-oriented perfectionism and two indices of psychological distress: depression and anxiety. In addition, socially-prescribed perfectionism was linked to depression, anxiety, social stress and aggressive behaviours.

Perfectionism has been extensively examined in the literature on giftedness [6], [7], [8], [9]. LoCicero and Ashby have explored various dimensions of perfectionism with gifted children (n = 83, m = 13 years old) and a group of non-gifted peers (n = 112) [10]. The authors used a questionnaire measuring personal standards of success (S) and the discrepancy between achieved performances and personal standards of success (D). They divided the distribution of “S” scores obtained into three classes of equivalent size. The perfectionists had the highest scores on S. The second dimension, D, was used to evaluate the adaptive character of perfectionism. The distribution of the D scores was split at the median forming two groups: adaptive perfectionism (lower half of the distribution) and maladaptative perfectionism (upper half of the distribution). The results indicate that the gifted obtained a higher mean score on S, which shows a tendency of this population to set higher criteria for success. In addition, the gifted show the weakest mean score on D, suggesting that high criteria of success are not necessarily a handicap for gifted children.

If perfectionism is a personality facet that can be useful for the expression of talent with high levels of accomplishment, it can also be associated with anxious feelings if one’s standards of accomplishment are never met. Thus, strong feelings and involvement, perfectionism, as well as non-challenging schoolwork and parental expectations, were relatively common socio-emotional problems encountered by gifted students [11]. In this view, gifted children may require particular support because of their strong tendency to be tense and anxious [12], but only a few empirical studies exist to support this point of view. For example, Roberts and Lovett [13] induced experimentally a failure situation in three groups of 20 teenagers, aged 12 to 14 years old: a group of gifted teenagers, a group of teenagers with good scholastic performance and a random control group from the general population. The results indicate a tendency for the gifted group to express more negative emotional and physiological reactions to stressors than the two other groups. However, all empirical research does not support this result; Roome and Roomney [14] conducted a study with 30 gifted children (11 to 14 years old) receiving a special educational program. Participants were evaluated on various psychological dimensions and compared to a representative group of children from the general population. No differences were found between these groups on measures of anxiety (trait and state). Furthermore, Scholwinski and Reynolds [15] presented the Children Manifest Anxiety Scale to a large sample of children and adolescents aged from 6 to 19 years old. Results showed lower levels of anxiety among subjects with high IQ.

These contradictory findings suggest that a specific context may favour manifestations of anxiety for gifted children. Perfectionist trait appears to be a plausible candidate as a component of this context. We propose to examine anxiety-related manifestations of higher levels of perfectionism on gifted pre-adolescent children.

Our first hypothesis is that significant positive correlations between measurements of anxiety and perfectionism should be observed.

Second, the literature on giftedness suggests that perfectionism personality traits are more salient among “gifted” populations. To test this hypothesis, we compared a group of gifted children with peers of 1) same scholar level and 2) same developmental level.

Third, we compared these groups on anxiety measures. If the previous hypotheses are verified, the gifted should also exhibit a higher level of anxiety.

## Methods

### Participants

The 132 participants of the study come from 3 schools located in the Parisian suburbs (France). A written parental consent was sent to parents. In this document, the aim and the design of the study were also explained. Only children with approved signed consent forms participated in the study.

The characteristics of the samples are summarized in table 1.

Melun (S^te^ Jeanne d’arc Institute, n = 64) : a semi-private establishment with gifted children in specific classes from the sixth grade onwards. The pupils are admitted after having been assessed on psychological tests measuring intellectual abilities, using Wechsler’s scales. The minimum required IQ level is 130.
Paris (Cours Hattemer, n = 18): a private establishment that receives gifted children. The gifted are identified using Wechsler’s intelligence scales (IQ>130).
Le Vésinet (Collège du Cèdre and « Les Merlettes » elementary school, n = 50) : this public school conducts collective assessments of cognitive abilities of children likely to have a high intellectual potential. Candidates pass a silent reading test for text comprehension and Cattell’s *Culture Fair Test*.

The originality of this study lies in the use of two control groups with respect to the gifted group investigated (see table 1). The gifted group is comprised of 6^th^ graders. Thus, the first control group includes non-gifted 6^th^ graders to match grade level, and the second control group includes non-gifted 5^th^ graders. Indeed, gifted children tend to be younger than their grade level peers, because of acceleration in their curriculum. The 5^th^ grade control group allows us to make comparisons with same age peers.

**Table 1:** Means and standard deviations of age for the 3 groups.

	*m*	*sd*	*n*
Gifted 6^th^graders	10.95	0.78	61
Non-gifted 6^th^graders	11.59	0.59	51
Non-gifted 5^th^graders	11.03	0.41	20

### Material

#### Measures of manifest anxiety

The RCMAS (*Revised Children and Adolescent Manifest Anxiety Scale*) [16] is a self-report questionnaire used to measure anxiety in children and adolescents from 6 to 19 years old. We used a 37 items validated French version [17]. The RCMAS, subtitled “What I think and Feel” consists of 28 Anxiety items and 9 Lie (social desirability) items. A high score indicates a high level of anxiety for each subscale. The RCMAS provides scores for Total Anxiety and four subscales: Physiological Anxiety: (10 items are associated with somatic manifestations of anxiety such as difficulties to fall asleep, nausea or fatigue); Worry/Oversensitivity (11 items related to obsessional concerns that are not clearly defined and are accompanied by fears of being affectively wounded or isolated); Social Concerns/Concentration (7 items linked to school difficulties, uncomfortable thoughts with a social or interpersonal component, difficulties of attention and concentration); Lie Scale (9 items to detect the tendency to consent, social desirability and falsification). In the French version, Cronbach’s alphas range from .59 (Physiological Anxiety) to .76 (Worry/Oversensitivity). The Total Anxiety’s alpha is .84.

#### Measures of perfectionism

The CAPS (*Children and Adolescent Perfectionism Scale*) [5] is a self-evaluation instrument designed to provide measures of a global score of perfectionism and two of its dimensions: self-oriented perfectionism (SOP) and socially-prescribed perfectionism (SPP). Participants express their level of agreement using a 5-point Likert scale (12 items for the self-oriented perfectionism subscale and 10 items for the socially-prescribed perfectionism subscale). The American version shows internal consistency of .86 and .85 for the two dimensions, respectively [18]. We applied a back translation method to construct a French version of the CAPS.

### Procedure

The questionnaires were part of a large evaluation that involved cognitive, socio-emotional and conative measurements. Children were seen during two sessions of 45mn each. The RCMAS was completed during the first session and the CAPS during the second one. Each child was tested individually, except for 5^th^ grade participants at Melun. For this sample, we observed abnormally high scores on perfectionism measures at a descriptive level (SPP score particularly). As the CAPS was administered collectively in the presence of the teacher, we suspected an effect of social desirability in children’s answers and decided to exclude them from the analyses.

Following recommandations of the test manual, we also excluded 16 participants whose scores on the Lie scale of the R-CMAS were above 12 points.

**Table 2:** Loadings on two factors resulting from a factor analysis with Varimax rotation of the Children and Adolescent Perfectionism Scale (reversed items are in italics).

	Self Oriented Perfectionism (21,03%)	Socially Prescribed Perfectionism (19,88%)
Item 1	0.59*	0.26
Item 2	0.64*	0.2
*Item 3*	*0*	*0.12*
Item 4	0.2	0.22
Item 5	0.16	0.71*
Item 6	0.57*	0.01
Item 7	0.68*	-0.06
Item 8	0.28	0.77*
*Item 9*	*0.4*	*0.13*
Item 10	0.07	0.55*
Item 11	0.65*	0.01
Item 12	0.19	0.73*
Item 13	0.23	0.77*
Item 14	0.68*	0.11
Item 15	0.22	0.80*
Item 16	0.72*	0.34
Item 17	0.39	0.25
*Item 18*	*0.24*	*0.11*
Item19	0.39	0.52*
Item 20	0.63*	0.1
Item 21	-0.02	0.71*
Item 22	0.68*	0.19

## Results

In the first part of the analyses, we provide descriptive statistics for our sample, and reliability of the CAPS French version. Then, we present correlational analyses between CAPS and RCMAS for the whole sample. Also we compare groups for both questionnaires. As the non-gifted 5^th^ grade group’s size is relatively small (20 children), we used a non-parametric statistical test (Mann-Whitney) for all comparisons that involve this group.

### Descriptive Statistics on the Sample

Our study involved three groups of children. There was a significant age difference between non-gifted 6^th^ graders and the gifted sample: gifted children were younger than their peers having the same scholastic level (t_(94)_ = 4.32; p<.001). No statistical differences on age were found between the gifted sample and children in 5^th^ grade (| z | = 0.69; p>.05).

### CAPS Reliability

The CAPS scale showed an acceptable reliability; Cronbach’s alpha was α = .87 for the total scale, α = .82 for the SOP scale and α = .84 for the SPP scale. A principal component analysis with Varimax rotation showed two factors that account for 40.91% of the variance. Table 2 shows items loadings by factors. The French version of CAPS exhibits a similar reliability and internal structure compared to the original one.

### Correlational Analysis

We present here regression analyses performed on the whole sample.

Table 3 shows a positive, significant correlation between the total score on CAPS and RCMAS, r = .35, p<.01. This result suggests a link between anxiety and perfectionism. Further analyses on CAPS and RCMAS dimensions provide more details on the nature of this link.

**Table 3:** Correlations between scores on Children and Adolescent Perfectionism Scale and the Revised-Children Manifest Anxiety Scale for the whole sample (n = 132).

	R-CMAS Total	Physiological	Worry/Oversensibility	Social concerns
CAPS Total	.35*	.10	.40*	.26*
Self Oriented Perfectionism	.25*	.04	.35*	.12
Socially Prescribed Perfectionism	.36*	.14	.34*	.34*
				* p<.01

First, SOP is positively linked to the Worry/Oversensibility dimension of RCMAS, with a significant correlation of.35 (p<.01). In contrast, only weak correlations were found with SOP with Social Concerns and the Physiological score (r = .12 and r = .04, respectively, p>.10). Second, SPP shows a positive correlation with the Worry/Oversensibility dimension, (r = .34, p<.01). However, this correlation is reduced to r = .13 (ns) after partialling out the SOP score. The correlation between SPP and Social Concerns is positive and significant, (r = .34, p<.01). Here again, the Physiological dimension of RCMAS is independent of CAPS total score (r = .14, p>.10).

### Gifted vs. Non-gifted: RCMAS

As shown in table 4, no significant differences were found between gifted and non-gifted 6th graders on RCMAS scores. Mann-Whitney tests show significant differences on certain subscales between gifted 6th graders and non-gifted 5th graders: the gifted tend to exhibit higher scores on Worry/Overexcitability (| z | = 1.73; p<.05 one-tailed) and Social Concerns (| z | = 2.08; p<.05). Differences on the RCMAS dimensions between non-gifted groups were not significant.

**Table 4:** Means and standard deviations of scores of anxiety measures for the experimental groups.

		Gifted 6^th^ graders (*n* = 61)	Non-gifted 6^th^ graders (*n* = 51)	Non-gifted 5^th^ graders (*n* = 20)
Total R-CMAS	m	52.64	52.33	48.55
	*sd*	*8.48*	*9.46*	*10.32*
Physiological	m	10.49	10.92	10.55
	*sd*	*2.48*	*3.81*	*2.87*
Worry/OversensibiIity	m	10.54	10.55	9.35
	*sd*	*2.67*	*2.90*	*3.08*
Social Concerns	m	10.69	10.73	9.55
	*sd*	*2.45*	*2.68*	*2.72*

### Gifted vs. Non-gifted: CAPS

CAPS showed differences between gifted and non-gifted 6th graders on the Total Score and SOP score. As presented in table 5, the mean Total Score for the gifted group is higher than the control group in 6th grade (t(110) = 2.28; p<.05). The same observation holds for the SOP score (t(110) = 2.37; p<.05). Furthermore, the SPP score failed to discriminate these two groups (t(110) = 1.50; p>.05).

**Table 5:** Means and standard deviations of scores of perfectionism measures for the experimental groups.

		Gifted 6^th^ graders (*n* = 61)	Non-gifted 6^th^ graders (*n* = 51)	Non-gifted 5^th^ graders (*n* = 20)
Total CAPS	m	67.43	60.86	70.30
	*sd*	*14.22*	*16.24*	*13.20*
Self Oriented Perfectionism	m	38.05	33.90	40.35
	*sd*	*9.17*	*9.24*	*5.73*
Socially Prescribed Perfectionism	m	29.38	26.96	29.95
	*sd*	*8.31*	*8.72*	*9.34*

This result was also observed when comparing the non-gifted groups, with a higher level of perfectionism for 5th graders, both on Total score (| z | = 2.06; p<.05) and SOP score (| z | = 2.66; p<.05). The SPP score does not show significant differences between these groups. No difference was found between the gifted group and non-gifted 5th graders on any CAPS scores.

## Discussion

The present study investigated the implication of perfectionism in intellectual giftedness and its possible links to manifest anxiety. Based on the literature on this issue, we hypothesized that intellectually gifted children would tend to show higher scores on perfectionism measures and that intellectually gifted children would express more anxiety than other children.

We found links between perfectionism and manifest anxiety which corroborate the results of Hewitt et al. [4]. The positive correlation between total scores on these scales suggests the possibility of an anxiety-producing effect of perfectionism. More specifically, SOP scores were associated with the worry/overexcitability dimension of the R-CMAS whereas SPP scores were associated with its social concern dimension. In related work, Mann [19] showed that SPP was significantly correlated with shame proneness and narcissistic injury that could be a source of anxiety in social interactions.

On one hand, we observed that 5^th^ graders tend to exhibit lower levels of anxiety compared to 6th graders. This could correspond to the transition between primary and secondary education. Among events children usually have to cope with, the modification of school environment is seen as one of the most stressful [20]. For example in French middle school, children must face changes they are not familiar with: dealing with a timetable, responding to the expectations of several teachers, or moving to different classrooms all along the day. They also have to live among much more students, and learn new social codes that are linked to the emergence of adolescence. Thus, their representation of school based on the experience of elementary school has to be revised and adjusted to a less containing environment, what could be a source of anxiety.

On the other hand, the perfectionism levels are higher for children in 5^th^ grade. Elementary school teachings (learning a recitation, writing lines of letters, memorizing multiplication tables) may favor the emergence of this obsessional personality trait. Furthermore, for these school levels, knowledge is transmitted by a sole teacher,. This could strengthen the desire to please teacher’s expectation and accentuate the willingness to appear perfect in order to fulfill it.

A paradox appears in our study with intellectually gifted children: this group expresses higher SOP scores than the control group in 6th grade whereas they show similar levels of anxiety. In contrast, gifted children showed the same level of perfectionism as non-gifted 5th graders, but expressed higher anxiety. This suggests that for the gifted, this personality trait may be more sensitive to internal development than to changes in school settings. From this point of view, the gifted are closer to their same-age peers than to their grade level peers. Thus the relationship between perfectionism and anxiety appears to be complex, and replications are needed as suggested by Sondergeld, Schultz and Glover [21]. Indeed, these interpretations are limited by the cross-sectional aspect of our study. An extension of this work would be to collect longitudinal data on the interplay between perfectionism and anxiety and/or depressive manifestations among gifted and non-gifted populations, in particular to distinguish between the impact of environmental factors and the role of developmental processes.

The results reported in the literature usually show lower levels of anxiety with intellectually gifted children compared to same-age groups [22]. In our study, as in the majority of studies in this area, gifted samples come from special education programs. Thus, these participants are already associated with a form of recognition, which constitutes another limitation of this work. It is possible that anxious behaviors are more likely to appear among gifted individuals whose potential remains unidentified. In this sense, the co-existence of a marked perfectionism and anxiety manifestations must to be taken into account by healthcare and educational professionals for the identification of gifted children. An extension of our study could involve distinguishing between healthy perfectionism and unhealthy perfectionism in gifted children. For this population, the use of a specific instrument for this clinical aspect could be useful to explore further the anxiety-producing side of perfectionism and prevent its possible evolution toward underachievement or psychiatric problems.

A third limitation of our study concerns the heterogeneity of our experimental groups. For example, the gifted group is composed of children who were identified using individual assessment (Weschler’s intelligence scales), whereas others through collective assessments (a silent reading test and Cattel’s Test). However, they share the distinction of being selected for their atypical performances on intellectual tasks.

Concerning the dimensions of perfectionism, intellectually gifted children showed higher SOP scores than the control group in 6^th^ grade, indicating a tendency to set higher criteria for success than their peers. However, similar scores on the SPP scale were observed for these two groups. In their investigation of perfectionism in sixth-grade students, Parker and Stumpf found only a small effect size for parental influence on perfectionism, accounting for less than 4% of the variance in children’s perfectionism scores [23]. This raises an important question on the origin of perfectionism traits observed in certain gifted populations. Is it a characteristic associated with high-level cognitive functioning that allows one to visualize the finished product of a task? If so, a potential source of anxiety could lie in the difficulties to plan and engage in intermediate steps that lead to this product. In this case, desire is transformed into demands, as formulated by Barrow and Moore (1983, see in Pyryt [24]). Another possibility is to view perfectionism as a social stereotype associated with giftedness in general. If so, the source of anxiety emerges from a willingness to conform to this stereotype and the over-investment of this personality trait. A third alternative has been suggested by Cook and Kearney [25]. They explored parental perfectionism and its internalization by youth. The results of their study indicate a specific link between SOP in mother and son: maternal SOP was most closely related to son’s SOP, whereas maternal SPP was most closely linked to son’s internalizing psychopathology. From this point of view, a specific family environment could favour the emergence of pathologic traits associated with perfectionism. Future research on giftedness should have an interest to clarify the importance of parental transmission of anxiety and perfectionism.

Another line of research that emerges from this study concerns the influence of school curriculum on the development of perfectionism. Providing data on the perfectionism-producing effect of school should be useful for educators who need to distinguish between regular and unhealthy perfectionism. It might also bring elements of reflection for choosing relevant options when it is required to build a curriculum acceleration plan for a student.
